# Pharmacological effects of baicalin in lung diseases

**DOI:** 10.3389/fphar.2023.1188202

**Published:** 2023-04-24

**Authors:** Duoning Wang, Yi Li

**Affiliations:** ^1^ Chengdu Hi-tech Nanxili Jiuzheng Clinic, Chengdu, Sichuan, China; ^2^ Department of Respiratory and Critical Care Medicine, Institute of Respiratory Health, Precision Medicine Key Laboratory, West China Hospital, Sichuan University, Chengdu, Sichuan, China

**Keywords:** baicalin, lung disease, lung infection, lung injury, lung cancer

## Abstract

The flavonoids baicalin and baicalein were discovered in the root of *Scutellaria baicalensis Georgi* and are primarily used in traditional Chinese medicine, herbal supplements and healthcare. Recently, accumulated investigations have demonstrated the therapeutic benefits of baicalin in treating various lung diseases due to its antioxidant, anti-inflammatory, immunomodulatory, antiapoptotic, anticancer, and antiviral effects. In this review, the PubMed database and ClinicalTrials website were searched with the search string “baicalin” and “lung” for articles published between September 1970 and March 2023. We summarized the therapeutic role that baicalin plays in a variety of lung diseases, such as chronic obstructive pulmonary disease, asthma, pulmonary fibrosis, pulmonary hypertension, pulmonary infections, acute lung injury/acute respiratory distress syndrome, and lung cancer. We also discussed the underlying mechanisms of baicalin targeting in these lung diseases.

## Introduction

**FIGURE 1:**
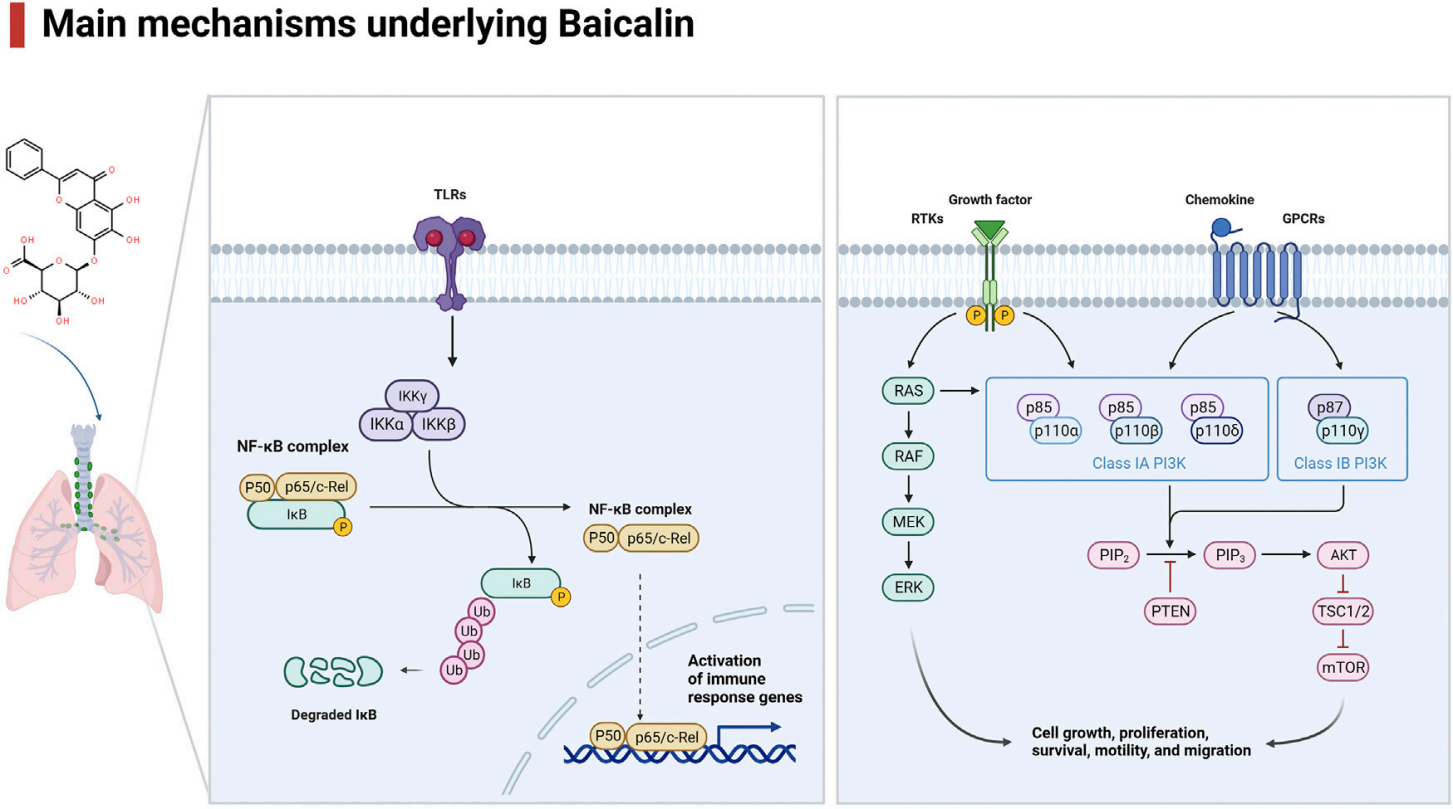
Regulatory signaling pathways implicated in baicalin.

**FIGURE 2:**
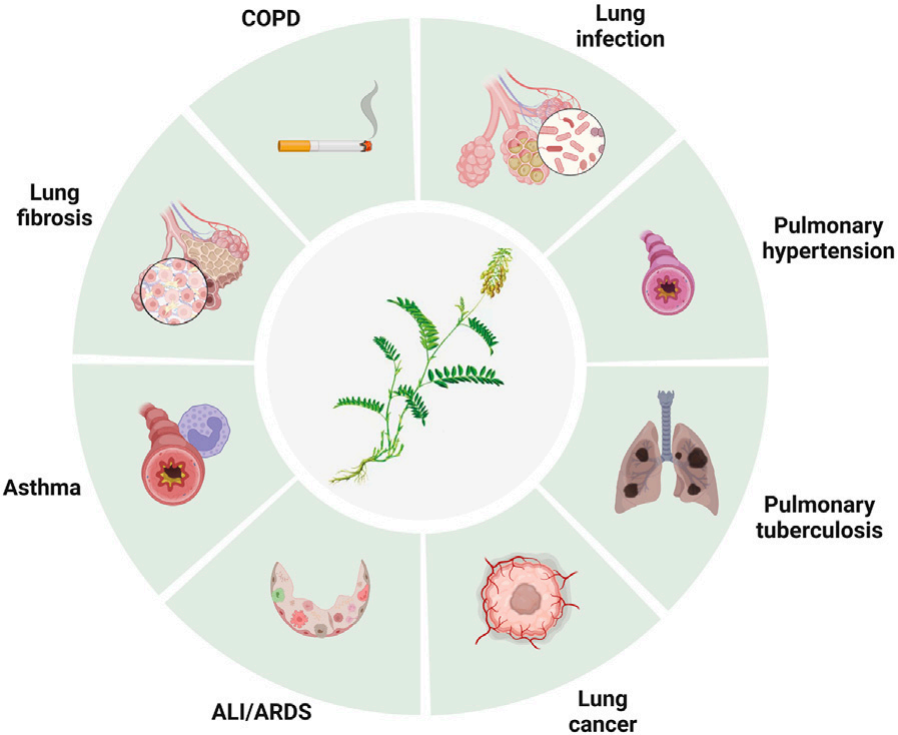
The applications of baicalin in lung diseases.

Lung disease is a major global health concern, affecting millions of people worldwide. Baicalin is a flavonoid compound isolated from the root of *Scutellaria baicalensis Georgi*. Baicalein is a flavone, a type of polyphenolic flavonoid, while baicalin is a flavone glycoside, the glucuronide of baicalein, which is obtained through the binding of glucuronic acid to baicalein. As a natural medicine, baicalin has thus been widely used in the treatment of clinical diseases such as cardiovascular and liver disease, diabetes, and neurodegenerative disorders. Baicalin has been shown to have potent antioxidant, anti-inflammatory, immunomodulatory, and antiapoptotic effects, making it a promising candidate for the treatment of several disease conditions. It thus has wide applications in medicine, healthcare, and food industries and has become a focused issue and trend in research worldwide in recent years. It has also been widely studied for its potential therapeutic benefits in treating various lung diseases, including chronic obstructive pulmonary disease (COPD), asthma and acute lung injury (ALI). The pharmacological contributions of baicalin to multiple lung diseases are being revealed (He et al., 2021) but are not fully understood. Baicalin exhibits anti-inflammatory and immunomodulatory effects by targeting several signaling pathways, including nuclear factor-κB (NF-κB), phosphatidylinositol-3-kinase (PI3K)/AKT), mitogen-activated protein kinases (MAPKs), and Toll-like receptors (TLRs), leading to a reduction in the production of proinflammatory cytokines and chemokines and subsequent development of inflammation in lung diseases (Figure 1, Table 1). In addition, baicalin has been found to have antioxidant and anti-apoptotic properties, which help prevent oxidative damage to cells and tissues. In addition, baicalin has been shown to have anticancer effects through its ability to inhibit the proliferation, migration, and invasion of cancer cells, including lung cancer cells, by inducing apoptosis and cell cycle arrest. Moreover, baicalin also exhibits antiviral effects against respiratory viruses such as influenza and severe acute respiratory syndrome coronavirus (SARS-CoV) by suppressing replication. In conclusion, baicalin shows great potential in the treatment of various lung diseases due to its anti-inflammatory, antioxidant, anticancer and antiviral properties. In this review, we will outline the recent understanding of baicalin treatment in lung diseases and its underlying mechanisms (Figure 2).

**TABLE 1:** Application of baicalin in experimental models of lung diseases.

Disease	Species	Model	Target/Pathway	References
COPD	Rat	CS	NF-κB↓	Lixuan et al. (2010)
	Mouse	CS	HDAC2 activity↑	Li et al. (2012)
	Rat	CS	Inflammatory cytokine regulation	Li et al. (2012), Wang et al. (2018)
	Rat	CS	HDAC2↑, NF-κB/PAI-1↓	Zhang et al. (2021a)
	Mouse	CS	HSP72↑, JNK↓	Hao et al. (2021)
	Rat	CS, LPS, cold stimulation	TLR2/MYD88/NF-κB p65↓	Ju et al. (2022)
	Mouse	PM2.5	Oropharyngeal microbiota balance	Deng et al. (2022)
			HMGB1/Caspase1↓	
Asthma	Mouse	OVA	ERK↓	Sun et al. (2013)
	Mouse	OVA	Th17 cells↓	Ma et al. (2014)
	Mouse	OVA + LPS	Th17/Treg balance	Xu et al. (2017a)
	Mouse	OVA	NF-κB, CCR7/CCL19/CCL21↓	Park et al. (2016)
	Mouse	OVA	miR-103↑, TLR4/NF-κB↓	Zhai and Wang (2022)
	Mouse	OVA	RAS↓	Hu et al. (2023)
PF	Mouse	Silica	Th17/Treg blance	Liu et al. (2015a)
	Rat	BLM	miR-21, TGF-β/Smad↓	Gao et al. (2013)
	Mouse	BLM	A2aR↑, TGF-β1, ERK1/2↓	Huang et al. (2016)
	Rat	BLM	GPX, SOD, GSH↑	Huang et al. (2016)
	Mouse	Radiation	CysLTs/CysLT1↓	Bao et al. (2022)
	Rat	BLM	SOD↑, MDA, HYP↓	Chang et al. (2021)
PH	Rat	Hypoxia	p38 MAPK/MMP-9↓	Yan et al. (2016)
	Rat	Hypoxia	AKT↑, HIF-1α↓, p27↑	Zhang et al. (2014)
	Rat	Hypoxia	ADAMTS-1↑, Collgen I↓	Liu et al. (2015b)
	Rat	MCT	NF-κB↓	Zhang et al. (2017a)
	Rat	MCT	TNF-α↓, BMPR2↑	Xue et al. (2021)
	Rat	MCT	AKT, eNOS↑, ERK, NF-κB↓	Yan et al. (2019)
Lung infection	Rat	*Pseudomonas aeruginosa*	Lung bacterial clearance↑	Zhang et al. (2021b)
	Mouse	*Staphylococcus aureus*	Lung microbial load↓	Brackman et al. (2011)
	Mouse	Streptozotocin-induced diabetes mellitus	Lung microbial dysbiosis	Wang et al. (2021a)
	Chicken	Avian pathogenic *Escherichia coli*	NF-κB↓	Peng et al. (2019)
	Chicken	*Mycoplasma* gallisepticum	TLR2/NF-κB↓	Wu et al. (2019)
	Chicken	*Mycoplasma* gallisepticum	Inflammatory injury alleviation	Wang et al. (2021b)
	Mouse	*Staphylococcus aureus*	Inflammatory injury alleviation	Liu et al. (2017)
	Mouse	*Mycoplasma* pneumoniae	miR-221, TLR4/NF-κB↓	Zhang et al. (2021c)
	Mouse	Influenza A/FM1/1/47(H1N1) virus	Lung virus titer↓	Xu et al. (2010)
	Rat			
	Mouse	H1N1 virus	Lung virus titer↓	Li and Wang (2019)
	Mouse	H1N1-H275Y	Neuraminidase activity↓	Jin et al. (2018)
	Mouse	Influenza A (H1N1/H3N2)	Neuraminidase↓	Ding et al. (2014)
	Mouse	H1N1-pdm09	NS1↓	Nayak et al. (2014)
	Mouse	Influenza A	TLR7/MYD88↓	Wan et al. (2014)
	Mouse	Influenza A	IFN-γ↑, JAK/STAT-1↑	Chu et al. (2015)
	Mouse	Influenza A	RLR↓	Pang et al. (2018)
	Mouse	Influenza A	Inflammatory cytokine regulation	Zhi et al. (2019)
	Mouse	Influenza A	macrophage M1 polarization	Geng et al. (2020)
			IFN↑	
	Mouse	RSV	Antiviral, anti-inflammatory	Shi et al. (2016)
ALI/ARDS	Mouse	Staphylococcal enterotoxin B	Tryptophan metabolism regulation	Hu et al. (2022)
	Mouse	CLP	HMGB1↓	Wang and Liu (2014)
	Rat	LPS	Inflammatory cytokine regulation	Huang et al. (2008)
	Mouse	LPS	NF-κB↓	Shin et al. (2015)
	Rat	SAP	Amylase, NO, MDA, TNF-α↓	Zhang et al. (2008)
	Mouse	SAP	TLR4↓	Li et al. (2009a)
	Mouse	LPS	TLR4/NF-κB↓	Zhang et al. (2021d)
	Mouse	LPS	CX3CL1-CX3CR1, NF-κB↓	Ding et al. (2016)
	Mouse	LPS	Nrf2/HO-1↑	Meng et al. (2019)
	Mouse	LPS	TLR4/NF-κB, JNK/ERK↓	Long et al. (2020)
	Rat	LPS	TLR4/MYD88/NF-Κb, MAPK↓	Changle et al. (2022)
	Rat	LPS	Alveolar fluid clearance, α-ENaC↑	Deng et al. (2017)
	Rat	LHP	Lipid-peroxidation in mitochondria↓	Liau et al. (2019)
	Rat	Air embolism	NF-κB↓	Li et al. (2009b)
	Rat	Severe burn	HMGB1, NLRP3, caspase-1, NF-κB, MMP-9↓	Bai et al. (2018)
	Mouse	Hyperoxia	Cpt1a↑	Chang et al. (2022)
	Mouse	LPS	TLR4/NF-κB, MMP-9↓	Zhang et al. (2016)
	Mouse	LPS	TLR4/p-NF-κB↓	Feng et al. (2018)
Lung cancer	Mouse	A549, LLC	HIF-1α↓, SOD↑	Du et al. (2010)
	Mouse	A549	Antitumor	Wei et al. (2017)
	Mouse	A549	Invasion, migration, angiogenesis↓	Yan et al. (2020)
	Mouse	H1299, H1650	Akt/mTOR↓	Sui et al. (2020)
	Mouse	A549, H1299	Id1↓	Zhao et al. (2019)
	Mouse	H460	EMT, PDK1/AKT↓	Chen et al. (2021)

CS: cigarette smoke; LPS: lipopolysaccharide; OVA: ovalbumin; BLM: bleomycin; MCT: monocrotaline; CLP: cecal ligation and puncture; SAP: severe acute pancreatitis; LHP: linoleic acid hydroperoxide.

## Methods

The PubMed database and ClinicalTrials website were searched for articles published between September 1970 and March 2023 using the following search string: (baicalin) and (lung). Studies of all designs that were accessible online were included if they met the following criteria: 1) published in English, 2) randomized control trials, other controlled trials, descriptive and comparative studies, evidence-based practice and 3) full-text available.

### COPD

The prevalence of COPD, which is currently the fourth largest cause of morbidity and mortality worldwide, is rising (Barnes et al., 2015; Barnes et al., 2019). Chronic airway inflammation, lung damage, and remodeling are its hallmarks, all of which lead to an irreversible blockage of airflow. Baicalin has been shown in studies to have anti-inflammatory and antioxidant effects that can help lessen the intensity of COPD symptoms. Moreover, baicalin has been shown to enhance lung health and lessen mucus formation in clinical practice, although there have been no reports to date. Additionally, it might protect the lungs from the harm caused by cigarette smoke and other environmental factors.

Baicalin has a variety of biological effects, including antioxidant and anti-inflammatory properties (Li et al., 2022). Mouse and cell models were stimulated by cigarette smoke (CS) and CS extract to investigate the effects and underlying mechanisms of baicalin on COPD. According to the findings, baicalin may control the balance between pro- and anti-inflammatory responses and significantly improve lung function in COPD patients (Lixuan et al., 2010; Li et al., 2012; Wang et al., 2018; Zhang et al., 2021a; Hao et al., 2021). The anti-inflammatory effect was probably caused by the inhibition of NF-κB activation (Lixuan et al., 2010), the upregulation of histone deacetylase 2 (HDAC2) activity (Li et al., 2012), and the modulation of the HDAC2/NF-κB/plasminogen activator inhibitor 1 (PAI-1) signaling pathways (Zhang et al., 2021a). Hao et al. (2021) demonstrated that baicalin upregulated the expression of heat shock protein 72 (HSP72), resulting in the inhibition of c-Jun N-terminal kinase (JNK) signaling activation and ultimately relieving COPD. Recently, Ju et al. (2022) proved that baicalin controlled the TLR2/myeloid differentiation primary response gene 88 (MYD88)/NF-κB p65 signaling pathway to reduce oxidative stress and the inflammatory response in COPD rats. Through regulating oropharyngeal microbiota and influencing the expression of the High Mobility Group Protein 1 (HMGB1)/Caspase1 pathway, baicalin may also mitigate mouse lung inflammatory injury caused by exposure to PM2.5 (Deng et al., 2022).

### Asthma

Asthma is a chronic inflammatory respiratory disorder that results in intermittent episodes of wheezing, breathlessness, chest stuffiness, and cough. Chronic bronchial inflammation, bronchial smooth muscle cell hypertrophy and hyperreactivity, as well as increased mucus output, are the disease’s defining characteristics (Papi et al., 2018).

In animal models of asthma, baicalin has been demonstrated to lower airway inflammation and enhance lung function (Sun et al., 2013), in part by modulating the Th17/Treg imbalance (Ma et al., 2014; Xu et al., 2017a). Additionally, baicalin has also been demonstrated to lessen mucus production in the airways and inhibit the contraction of bronchial smooth muscle, which might lessen airway narrowing and improve breathing (Xu et al., 2017a). In a previous study, baicalin pretreatment inhibited the MAPK signaling pathway, significantly reducing the proliferation and migration of airway smooth muscle cells (ASMCs) stimulated by platelet-derived growth factor (PDGF) (Yang et al., 2015). Baicalin administration reduced inflammatory cell infiltration and tumor necrosis factor-α (TNF-α) levels in bronchoalveolar lavage fluids in an animal model of allergic asthma, demonstrating that the anti-inflammatory actions of baicalin *in vivo* are due to its capacity to inhibit phosphodiesterase 4 (PDE4) (Park et al., 2016). By inhibiting NF-κB and reducing CC-chemokine receptor 7 (CCR7)/C-C motif chemokine ligand 19 (CCL19)/CCL21, Liu et al. showed that oral treatment with baicalin greatly enhanced pulmonary function and reduced inflammatory cell infiltration into the lungs (Liu et al., 2016), and Zhai et al. found that baicalin increased miRNA-103 and mediated the TLR4/NF-κB pathway to successfully reverse ovalbumin (OVA)-induced oxidative stress, inflammation, and changes in the amount of total cells, eosinophils, and neutrophils in bronchoalveolar lavage fluid (BALF) as well as collagen deposition (Zhai and Wang, 2022).

Baicalin significantly decreased the infiltration of inflammatory cells in lung tissue, attenuated airway resistance, and reduced the levels of remodeling-related cytokines such as interleukin (IL-13), vascular endothelial growth factor (VEGF), transforming growth factor-*β*1 (TGF-β1), matrix metalloproteinase 9 (MMP9), and tissue inhibitor of metallopeptidase 1 (TIMP1) at both the mRNA and protein levels. In an OVA-induced asthmatic mouse model, baicalin administration suppressed the RAS signaling pathway to prevent airway remodeling and ASMC proliferation by regulating the activation of protein kinase C-α (PKC-α), A-rapidly accelerated fibrosarcoma (A-RAF), mitogen activated protein kinase 2 (MEK2), extracellular regulated MAP kinase (ERK), MAPK interacting serine/threonine kinase 1 (MNK1), and ETS transcription factor 1 (ELK1) (Hu et al., 2023). Baicalin has recently been demonstrated to inhibit type 2 immunity by severing the interaction between mast cells and airway epithelial cells, suggesting that it may be a useful alternative therapy for the management of asthma (Yoshida et al., 2021). Baicalin may therefore be an effective medication for treating allergic and asthmatic disorders in humans by regulating NF-κB activity and other signaling pathways.

### Pulmonary fibrosis

Pulmonary fibrosis (PF) is a progressive, resistant pulmonary fibrotic condition with no known cause. Patchy but progressive bilateral interstitial fibrosis is the defining feature of PF, and in advanced cases, it can cause severe hypoxia and cyanosis (Lederer et al., 2018).

Treatment with baicalin in a mouse model of silicosis reduced the buildup of inflammatory cells by balancing the Th17 and Treg responses, which also resulted in fewer clinical inflammatory and fibrotic alterations in lung tissues (Liu et al., 2015a). Baicalein oral administration significantly reduced miR-21 levels, increased TGF-β1 and p-smad2/3 expression, and decreased hydroxyproline content and α-smooth muscle actin (α-SMA) levels in lung tissue, which is important for myofibroblast activation and collagen deposition in the extracellular matrix (Gao et al., 2013). In a different study, Huang et al. hypothesized that baicalin exerts its antifibrotic effects by modulating the expression of the adenosine A2a receptor (A2aR) gene, which regulates inflammation by lowering the levels of increased TGF-β1 and p-ERK1/2 (Huang et al., 2016). Subsequent research showed that baicalin considerably boosted serum levels of glutathione peroxidase (GPX), superoxide dismutase (SOD), and glutathione (GSH) while significantly lowering serum levels of malondialdehyde (MDA). Baicalin also controlled cyclin A, D, and E, proliferating cell nuclear antigen, p-AKT, and p-calcium/calmodulin dependent protein kinase type, suppressing the transition of cells from the G0/G1 phase to the G2/M and S phases and lowering the intracellular Ca^2+^ concentration to suppress bleomycin-induced pulmonary fibrosis and fibroblast proliferation (Zhao et al., 2020). By modulating the TGF-β and ERK/glycogen synthase kinase (GSK3β) signaling pathways (Lu et al., 2017), as well as the cysteinyl leukotriene (CysLTs)/CysLT1 pathway (Bao et al., 2022), baicalin alleviates radiation-induced epithelial-mesenchymal transition of primary type II alveolar epithelial cells. Hong et al. revealed the antifibrotic mechanisms of baicalin, which involve the regulation of four key biomarkers involved in the metabolism of taurine, hypotaurine, glutathione and glycerophospholipids (Chang et al., 2021). Studies have shown that baicalin treatment can improve lung function and reduce symptoms; thus, it may be a useful alternative therapy for the management of PF.

### Pulmonary hypertension

Pulmonary hypertension (PH) is usually secondary to a reduction in vessel diameter or an increase in blood flow in the pulmonary vascular bed (Vonk Noordegraaf et al., 2016). Most kinds of PH are thought to have a potential basis in the malfunctioning of pulmonary endothelial cells and/or vascular smooth muscle cells. The entire pulmonary arterial tree thickens the intima and media while narrowing the lumen as a result of endothelial and smooth muscle cell proliferation (Cui et al., 2022). Pretreatment with baicalin in chronic hypoxic rats attenuated PH and right-sided heart dysfunction by reducing p38 MAPK activation, reducing the elevated levels of the proinflammatory cytokines IL-1, IL-6 and TNF-α and downregulating the expression of MMP9 in the pulmonary arteriole walls (Yan et al., 2016).

Baicalin decreased hypoxia inducible factor-1 (HIF-1) production in a model of hypoxia-induced PH by modulating the AKT signaling pathway to stop p27 degradation. Increased p27 levels thereby inhibited pulmonary artery smooth muscle cell (PASMC) proliferation, preventing hypoxia-induced increased pulmonary arterial pressure and pulmonary vascular remodeling (Zhang et al., 2014). Another study found that baicalin suppressed the HIF-1α and aryl hydrocarbon receptor (AhR) pathways, which prevented TGF-β1-induced phenotypic switching and consequently the excessive growth of pulmonary arterial smooth muscle cells (Huang et al., 2014). One study indicated that baicalin provided protection for rats suffering from hypoxic PH. The mechanism may involve an increase in ADAM metallopeptidase with thrombospondin type 1 motif 1 (ADAMTS-1) expression, which inhibits collagen I synthesis and expression (Liu et al., 2015b).

By inhibiting the inflammatory response and downregulating the NF-κB signaling pathway, baicalin can considerably lower the expression of TGF-β1 in lung tissues and pulmonary arterial pressure, lessen right ventricular hypertrophy and injury, and attenuate pulmonary vascular remodeling (Luan et al., 2015). Baicalin has been found in numerous studies to significantly reduce P38 MAPK and MMP-9 expression. It effectively improved hypoxia-induced PH in a rat model by blocking the p38 MAPK signaling pathway and MMP-9 in the small pulmonary arteries (Yan et al., 2016).

An earlier study revealed that baicalin had a therapeutic effect on the hypoxia-induced PH rat model, at least in part because it activates peroxisome proliferator-activated receptor γ (PPARγ) and blocks the HMGB1/receptor for advanced glycation end-products (RAGE) inflammatory signaling pathway (Chen and Wang, 2017). In another study, baicalin exhibited increased A2aR activity and decreased stromal cell derived factor-1 (SDF-1)/C-X-C motif chemokine receptor 4 (CXCR4)-induced PI3K/AKT signaling to protect against hypoxia-induced PH (Huang et al., 2017).

Baicalin can inhibit monocrotaline (MCT)-induced PH in rats by upregulating bone morphogenetic protein 4 (BMP4), BMP9, BMP receptor 2 (BMPR2) and p-Smad1/5/8 expression, according to recent research. Baicalin greatly reduces the expression of NF-κB, TNF-α, IL-6 and IL-1. Baicalin, on the other hand, may reduce pulmonary vascular remodeling by preventing ERK and NF-κB phosphorylation and expression, as well as by suppressing endothelial-to-mesenchymal transition (EndMT) via the BMP/Smad axis and NF-κB signaling (Zhang et al., 2017a; Xue et al., 2021). By decreasing p-p65 and p-ERK expression and encouraging p-AKT and p-endothelial nitric oxide synthase (eNOS) expression, baicalin ameliorated pulmonary vascular remodeling and cardiorespiratory injury in the development of pulmonary arterial hypertension through the AKT/eNOS, ERK and NF-κB signaling pathways (Yan et al., 2019; Xue et al., 2021). By boosting the expression of ADAMTS-1, which inhibits the synthesis of type I collagen and its mRNA expression, baicalin administration significantly decreased pulmonary artery pressure and slowed the remodeling of the pulmonary artery under hypoxic conditions, according to Liu et al. (Liu et al., 2015b).

### Pulmonary infections

Pneumonia-related deaths from lung infections are common worldwide (McAllister et al., 2019). The lung’s epithelial surfaces are constantly exposed to microbial pollutants in the open air, and other frequent lung conditions and bad lifestyle choices, such smoking and drinking, make the lung parenchyma susceptible to pathogenic organisms.

Treatment with baicalin considerably lessened the severity of lung pathology and sped up the clearance of *Pseudomonas aeruginosa* from the lungs. After baicalin treatment, the Th1-induced inflammatory response and decreased cell infiltration in the lung around the implants were observed (Zhang et al., 2021b). In a different investigation, tobramycin treatment in combination with baicalin hydrate reduced the microbial load in mouse lungs infected with Burkholderia cenocepacia more than tobramycin treatment alone (Brackman et al., 2011). Via an NF-κB signaling pathway, baicalin might also treat the microbial dysbiosis of the lungs and the subsequent fibrogenesis in streptozotocin-induced diabetic mice (Wang et al., 2021a). Baicalin has also been shown to control the same pathway in avian pathogenic *Escherichia* coli-induced acute lung injury (Peng et al., 2019). Baicalin alleviates lung inflammatory injury in *Mycoplasma* gallisepticum infection models by inhibiting the TLR2/NF-κB pathway (Wu et al., 2019) and regulates gut microbiota and phenylalanine metabolism by modulating the gga-miR-190a-3p-Fas-associated death domain (FADD) axis in HD11 macrophages (Wang et al., 2021b). Baicalin also inhibits the development of *Staphylococcus aureus* pneumonia (Liu et al., 2017).

Among chronic pneumonias, pulmonary tuberculosis is a dangerous infectious illness that poses a substantial threat to human health. According to the World Health Organization, it kills 6% of people worldwide and is now getting worse. Th1 cells are primarily responsible for driving immunity against tuberculosis infection by causing macrophages to kill bacteria (Jasenosky et al., 2015). Re-exposure to *Mycobacterium tuberculosis* (Mtb) or the reactivation of the infection in a previously sensitized host triggers a rapid defense response, although hypersensitivity also accelerates tissue necrosis and destruction. The findings showed that baicalin did not affect the phosphorylation of p38, JNK, or ERK in either Raw264.7 or primary peritoneal macrophages but did decrease the levels of p-AKT and p-mammalian target of rapamycin (mTOR) at Ser473 and Ser2448, respectively. Moreover, baicalin increased the colocalization of inflammasomes with autophagosomes to exert an autophagic degradative effect on reducing inflammasome activation and exerted an inhibitory effect on NF-κB activity. Via the PI3K/AKT/mTOR pathway, baicalin causes autophagy activation in Mtb-infected macrophages. Moreover, baicalin inhibited the PI3K/AKT/NF-κB signaling pathway, and both autophagy induction and NF-κB inhibition contributed to limiting the activity of the NOD-like receptor thermal protein domain associated protein 3 (NLRP3) inflammasome and the resultant release of the proinflammatory cytokine IL-1β (Zhang et al., 2017b).

Another study showed that baicalin might limit protein kinase R-like endoplasmic reticulum kinase (PERK)/eukaryotic translation initiation factor 2 (eIF2) pathway activation, which would then downregulate thioredoxin interacting protein (TXNIP) expression and reduce the activation of the NLRP3 inflammasome, resulting in reduced pyroptosis in macrophages with Mtb infection (Fu et al., 2021). Baicalin relieves *Mycoplasma* pneumoniae infection-induced lung injury by blocking miRNA-221 to regulate the TLR4/NF-κB signaling pathway (Zhang et al., 2021c).

Baicalin exhibits inhibitory effects on various strains of influenza virus and SARS-CoV, both *in vitro* and *in vivo* (Limanaqi et al., 2020). Oral administration of baicalein to mice infected with the influenza virus increased the average survival time, reduced lung inflammation, and dose-dependently decreased the lung viral titer. These effects are probably caused by baicalin, which has been demonstrated to impede the replication of SARS-CoV and influenza virus *in vitro* (Chen et al., 2004; Xu et al., 2010). Via the TNF receptor associated factor 6 (TRAF6)-dependent production of Type-I interferons (IFNs), baicalin has been demonstrated to suppress influenza virus replication, which correlates with protection from acute lung injury in infected mice (Li and Wang, 2019). This is noteworthy because, in SARS-CoV, similar to what was observed in the influenza virus, alterations in mitochondrial homeostasis and autophagy were eventually caused by abnormal ubiquitin proteasome system (UPS)-dependent degradation, which was related to the suppression of inhibition of TRAF6-dependent expression of Type-I IFNs (Shi et al., 2014).

Baicalin suppresses influenza virus infection both *in vitro* and *in vivo* by directly inhibiting the neuraminidase surface glycoprotein, which is necessary for viral replication and the release of virions from infected cells (Ding et al., 2014; Jin et al., 2018). While baicalein strengthens the antiviral activity of the neuraminidase inhibitor zanamivir, sodium baicalin is also effective against oseltamivir-resistant mutant influenza virus strains (Sithisarn et al., 2013). The anti-neuraminidase activity of baicalein is accompanied by a reduction in TNF-α, IL-6, and IL-8, which is associated with inhibition of the NF-κB and PI3K/AKT pathways and may indicate autophagy is being activated (Sithisarn et al., 2013). This makes sense given that baicalin has been demonstrated to directly target the NS1 protein of the influenza virus, which has been proven to impair autophagy by activating PI3K/AKT (Nayak et al., 2014). Baicalin inhibited virus replication and reduced the activity of major factors of the RIG-I-like receptor (RLR) signaling pathway components, including retinoic-acid-inducible gene I (RIG-I), IFN regulatory factor 3 (IRF3), IRF7, NF-*κ*B, inflammatory responses, and macrophage polarization, in an influenza A virus infection model (Wan et al., 2014; Chu et al., 2015; Zhu et al., 2015; Pang et al., 2018; Zhi et al., 2019; Geng et al., 2020). Treatment with baicalin can also moderately lower respiratory syncytial virus titers recovered from lung tissues with a decrease in T lymphocyte infiltration and proinflammatory factor gene expression (Shi et al., 2016).

The pathologic mechanism of the coronavirus disease (COVID-19) outbreak caused by SARS-CoV-2 is still not completely clear (Huang et al., 2020; Wang et al., 2020). The most common cause of death in severe COVID-19 cases is respiratory failure, and conditions such as acute respiratory distress syndrome (ARDS), septic shock, severe metabolic acidosis, and a hypercoagulable state can be fatal. Baicalin, herbacetin, and pectolinarin have been found to effectively inhibit the proteolytic activity of the main protease, 3-chymotrypsin-like protease (3CLpro), and show effective inhibitory activity against SARS-CoV-2 3CLpro (Jo et al., 2020). It was predicted that baicalin would bind to papain-like protease (PLpro) (Lin et al., 2021) and the S protein (Boozari and Hosseinzadeh, 2021) with considerable affinity. Baicalin is an intriguing prospective therapeutic candidate for future study against SARS-CoV-2 that has been shown to bind the N-terminus and C-terminus of the homology model of the SARS-CoV-2 proteins non-structural protein 14 (Nsp14) and 3CLpro (Su et al., 2020; Liu et al., 2021). Baicalin and ascorbic acid can work together to reduce SARS-CoV-2 entrance by inhibiting the production of angiotensin-converting enzyme II in human small alveolar epithelial cells (Lai et al., 2021). When used to treat COVID-19, certain traditional Chinese medications containing baicalin have been shown to have immunological modulation, anti-infection, anti-inflammation, and multiorgan protection mechanisms (Su et al., 2020; Zhao et al., 2021a; Huang et al., 2022; Wei et al., 2022).

### Acute lung injury/acute respiratory distress syndrome

Damage to the alveolar capillary membrane, which is made up of the microvascular endothelium and the alveolar epithelium, results in pulmonary infiltrates in ALI. In the presence of sepsis, severe trauma, or extensive lung infection, ALI can progress to ARDS, which is more serious diffuse alveolar injury (Matthay et al., 2012).

By regulating the composition of the gut microbiota, increasing the production of short-chain fatty acids, and altering the fecal metabolite profiles via the lung-gut axis, baicalin can ameliorate staphylococcal enterotoxin B-induced ARDS (Hu et al., 2022). Baicalin inhibits the release of HMGB1 and cytokines from macrophages, improves survival and reduces tissue injury in septic mice induced by lipopolysaccharide (LPS) (Wang and Liu, 2014). By inhibiting TLR4, baicalin has a therapeutic impact on LPS-induced ALI (Huang et al., 2008; Shin et al., 2015) as well as acute pancreatitis-associated lung injury (Zhang et al., 2008; Li et al., 2009a; Zhang et al., 2021d). Ding et al. (2016) explained the crosstalk between the C-X3-C motif chemokine ligand 1 (CX3CL1)- C-X3-C motif chemokine receptor 1 (CX3CR1) axis and NF-κB pathway, while Meng et al. revealed that oxidative stress and inflammation were reduced via the activation of the nuclear factor erythroid 2-related factor 2 (NRF2)-mediated heme oxygenase 1 (HO-1) signaling pathway (Meng et al., 2019). Meanwhile, Long and Zhu et al. demonstrated that the mechanism involves the inhibition of the TLR4/NF-*κ*B p65 and ERK/JNK signaling pathways (Long et al., 2020) as well as the TLR4/MyD88/NF-κB/NLRP3 signaling pathway and the MAPK signaling pathway (Changle et al., 2022). Duan et al. (2021) demonstrated that it can attenuate follistatin-like protein 1 (FSTL1) and the ERK/JNK signaling pathway by upregulating miR-200b-3p expression. It may also prevent LPS-induced reduction of alveolar fluid clearance by upregulating epithelial sodium channel α-epithelial sodium channel (α-ENaC) protein through activation of the cyclic adenosine monophosphate (cAMP)/protein kinase A (PKA) signaling pathway to attenuate lung edema (Deng et al., 2017). Another investigation demonstrated that baicalin can reduce lung mitochondrial lipid peroxidation and antioxidant activity induced by linoleic acid hydroperoxide (LHP) *in vitro* (Liau et al., 2019).

Baicalin attenuated air embolism-induced acute lung injury (Li et al., 2009b) and severe burn-induced remote acute lung injury through the NLRP3 signaling pathway (Bai et al., 2018) and attenuated neonatal hyperoxia-induced endothelial cell dysfunction and alveolar and vascular simplification in adult mice by upregulating carnitine palmitoyltransferase 1a (Cpt1a) (Chang et al., 2022). These results suggest possible treatment approaches for employing baicalin to prevent persistent lung injury in some illnesses and injuries.

Pharmacological studies have shown suppression of TLR4-mediated NF-κB activation (Zhang et al., 2016; Feng et al., 2018), downregulation of MMP9 expression (Zhang et al., 2016), inhibition of p-Src in LPS-activated neutrophils and formation of neutrophil extracellular traps (NETs) in Phorbol 12-myristate 13-acetate (PMA)-induced neutrophils (Xiao et al., 2022) in response to traditional Chinese medications containing baicalin.

### Lung cancer

Baicalin has inhibitory effects on the proliferation and migration of various tumor cells. It can promote tumor cell apoptosis through multiple pathways and enhance the effectiveness of chemotherapy and radiotherapy (Zhang et al., 2017c; Singh et al., 2021). Baicalin can prevent lung cancer cells from growing and spreading by inducing apoptosis and cell cycle arrest and inhibiting the production of proinflammatory and protumor cytokines, according to research conducted on animals and in cell culture (Mizushima et al., 1995; Cheng et al., 2003; Himeji et al., 2007; Du et al., 2010; Jangid et al., 2020), and formulations and derivatives are emerging (Zhang et al., 2017c; Li et al., 2017; Wei et al., 2017; Jangid et al., 2020). By activating p38 MAPK and generating intracellular reactive oxygen species, baicalin enhances TNF-related apoptosis-inducing ligand (TRAIL)-induced apoptosis (Zhang et al., 2017c).

The antitumor effects of baicalin mainly include inhibiting the proliferation of tumor cells by blocking the cell cycle, inducing apoptosis in tumor cells by producing cytotoxicity, and suppressing the erosion and metastasis of tumor cells (Sui et al., 2020; Yan et al., 2020). According to reports, baicalin can block cell cycle progression in the S phase by suppressing cyclin A, but in SKLU1, SKMES1 and DU145 cells, baicalin suppresses the expression of cyclin D1, leading to cell cycle arrest in the G1 phase (Gao et al., 2011). Baicalin stimulates the sirtuin 1 (SIRT1)/AMP-activated protein kinase (AMPK) signaling pathway (You et al., 2018), inhibits p-AKT in tumor cells, and attenuates cisplatin resistance in lung cancer by downregulating MARK2 and p‐AKT (Xu et al., 2017b). Yin et al. reported that baicalin attenuates X-ray cross complementing 1 (XRCC1)-mediated DNA repair to enhance the sensitivity of lung cancer cells to cisplatin. Baicalin activated Rap1-GTP binding and dephosphorylated AKT and Src by suppressing a7 nicotinic acetylcholine receptor (a7nAChR), consequently triggering inhibition of inhibitor of differentiation factor 1 (Id1) (Zhao et al., 2019). Diao et al. demonstrated that baicalin inhibits lung cancer growth by targeting PDZ-binding kinase/T-LAK cell-originated protein kinase (PBK/TOPK) (Yan et al., 2020), while Chen et al. revealed that baicalin prevents epithelial-mesenchymal transition (EMT) by blocking the pyruvate dehydrogenase kinase 1 (PDK1)/AKT pathway in human non-small cell lung cancer (NSCLC) (Chen et al., 2021). Baicalin’s antitumor effects were shown by Zhao et al. to be mediated through the miR-340-5p-neuroepithelial cell transforming 1 (NET1) axis (Zhao et al., 2021b). Follow-up research can carry out more effective and precise interventions in the above and other regulatory pathways, inhibiting the growth and metastasis of lung cancer cells and prolonging the survival of patients.

## Conclusion and future directions

The evidence thus far suggests baicalin may be a promising option for managing symptoms and preventing disease progression of various lung diseases due to its anti-inflammatory, antioxidant, anticancer and antiviral properties, although research on baicalin as a treatment for lung disease is still ongoing. While research on baicalin for the treatment of lung disease holds potential, there are still limitations to be addressed. First, many of the studies have focused on animal models; thus, more human clinical trials are needed to evaluate the efficacy and safety of baicalin in treating lung diseases. Baicalin has been found to be a safe and well-tolerated treatment for lung disease with a low incidence of adverse effects. Further research is needed to confirm its long-term safety and therapeutic benefits and to optimize its use as a treatment for lung disease. Additionally, the optimal dose and duration of treatment need to be determined, as well as the potential for drug interactions with other medications. With greater research into its mechanisms of action, long-term effects, application domains, and the continual expansion of its application, its value will continue to be unearthed and investigated.
